# Fabrication of Si/graphene/Si Double Heterostructures by Semiconductor Wafer Bonding towards Future Applications in Optoelectronics

**DOI:** 10.3390/nano8121048

**Published:** 2018-12-14

**Authors:** Takenori Naito, Katsuaki Tanabe

**Affiliations:** Department of Chemical Engineering, Kyoto University, Kyoto 615-8510, Japan; naito@cheme.kyoto-u.ac.jp

**Keywords:** graphene, silicon, double heterostructure, wafer bonding, optoelectronics, nanophotonics

## Abstract

A Si/graphene/Si planar double heterostructure has been fabricated by means of semiconductor wafer bonding. The interfacial mechanical stability and interlayer electrical connection have been verified for the structure. To the best of our knowledge, this is the first realization of a monolayer-cored double heterostructure. In addition, a double heterostructure with bilayer graphene has been prepared for bandgap generation and tuning by application of a bias voltage. These structures move towards the realization of versatile graphene optoelectronics, such as an electrically pumped graphene laser. Our Si/graphene/Si double heterostructure is positioned to form a new basis for next-generation nanophotonic devices with high photon and carrier confinements, earth abundance (C, Si), environmental safety (C, Si), and excellent optical and electrical controllability by silicon clads.

## 1. Introduction

Monolayer-material-based optoelectronic devices possess promising characteristics for high-density integration, low-power-consumption, and high-speed operation [1,2,3,4,5,6,7,8,9,10,11]. However, monolayer materials have been used nakedly in the devices reported so far, and such devices suffer from a substantial amount of electrical and optical losses. To address this issue, we have employed monolayer-cored double heterostructures [12,13], which allow for carrier and photon confinements. Monolayer-gained semiconductor lasers show promise in the nanophotonics field [14,15]. Graphene lasers, if realized, would be an ultimate component in optoelectronics in view of environmental friendliness, potential low cost, small scale for high-density integration, and superb physical properties [3,7,9]. Semiconductor wafer bonding [16,17,18,19,20,21,22] is utilized to form heterostructures of dissimilar semiconductor materials with low defect densities, which is otherwise difficult to obtain by the conventional growth method due to the crystalline lattice mismatch. Wafer bonding is therefore promising for fabrication of high-performance semiconductor optoelectronics, and has been employed to generate a variety of heterostructured devices [23,24,25,26,27,28,29]. Here, we have fabricated a Si/graphene/Si double heterostructure by graphene-mediated bonding, serving as the first preparation of a monolayer-cored double heterostructure capable of providing a future basis for high-performance nanophotonic devices.

## 2. Experimental Methods

We used commercially available monolayer and bilayer graphene materials (Graphene Platform Corporation, Yokohama, Japan) in this work. Silicon, a versatile semiconductor, was adopted as the cladding material. We used single-side polished, epi-ready *p*-type Si <100> wafers doped with boron (doping concentration of ~1 × 10^19^ cm^−3^) and double-side polished, epi-ready *p*-type Si <100> wafers doped with boron (doping concentration of ~1 × 10^16^ cm^−3^). Large-area graphene sheets (~20 cm^2^) transferred onto the polished-side surface of the Si wafers was used in this study. In an attempt to create balance between ease in obtaining electric measurements from ohmic metal contacts and optical transparency for transmission measurements, the higher and lower doping-concentration Si wafers were used for monolayer and bilayer graphene, respectively. The Si wafer topped with a graphene layer was cut into ~1 cm^2^ area dies after being coated with a photoresist, in order to protect the bonding surface from particles generated during the cutting process. During cutting, no issues were observed for the graphene layer, which remained intact on the Si wafer and did not experience delamination. A bare Si wafer was also coated with a photoresist and then cut into ~0.64 cm^2^ area dies. Immediately before bonding, the photoresist on both dies was removed with acetone, along with degreasing of the bonding surfaces. The graphene-side surface of the graphene-on-Si wafer was brought into contact with the polished-side surface of a bare Si wafer with the same doping concentration, with their Si (011) edges aligned. The two die pieces were then bonded by annealing in ambient air for 3 h under a uniaxial pressure of 0.1 MPaG [28]. The detachment normal stresses were measured as the bonded interfacial strengths for the fabricated Si/graphene/Si samples, as well as Si/Si direct-bonded controls (no graphene). For current injection during electrical measurements, Al layers with a thickness of 100 nm were deposited by electron-beam evaporation as ohmic electrodes on both outer Si surfaces of the bonded samples containing a higher doping concentration. Alternatively, metal electrodes comprising an Au-Ge-Ni alloy (80:10:10 wt%) and pure Au with thicknesses of 30 and 150 nm, respectively, were sequentially deposited by thermal evaporation on the samples containing a lower doping concentration.

**Figure 1:**
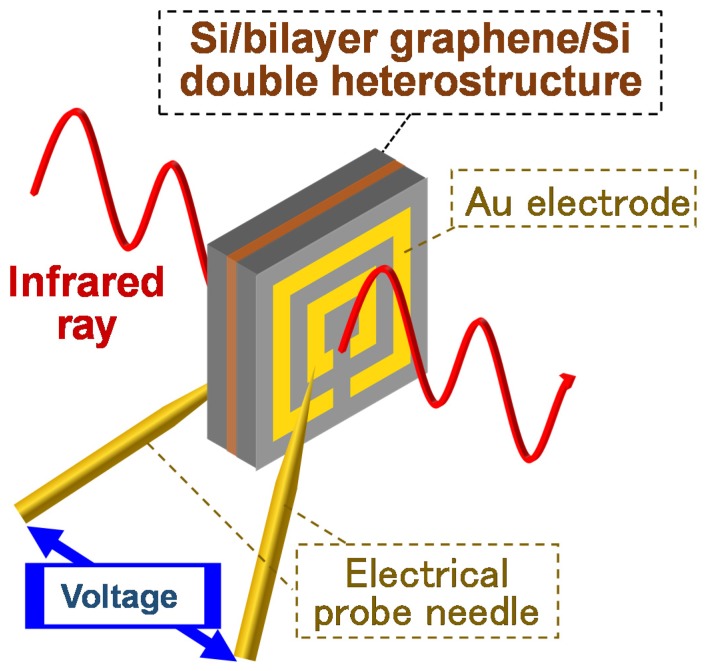
Conceptual drawing of the measurement system used for graphene bandgap tuning and detection.

In the second part of this work, the functionality of the structure was demonstrated by attempting to generate and tune the graphene bandgap energy through application of an electrical bias voltage. Bilayer graphene [30] was used as the core material in the Si-cladded double heterostructure. Graphene bandgap energy is known to be detectable via observation of absorption peaks in transmission spectra [30]. According to Reference [30], bias voltages were applied in the normal direction of the planar Si/bilayer graphene/Si double heterostructures, and the measured optical transmission in the normal direction vertically penetrated the samples. Figure 1 depicts a conceptual drawing of the measurement system. Optical measurements utilizing Fourier-transform infrared spectroscopy (FTIR, PerkinElmer, Waltham, MA, USA) were conducted to observe the graphene bandgap. Attempts were made to detect the existence of the absorption peak induced by the bandgap energy, by eliminating optical interference from the acquired transmission spectral data using the after-mentioned theoretical calculations. Due to facility limitations, measurements were conducted in ambient air at room temperature in the photon energy range from 0.05 to 0.85 eV and applied bias voltage from −20 to 20 V. To clarify the change in transmission spectra induced by the bias voltage, the raw transmission spectra data obtained with bias voltage, *V*, were normalized to that without electrical bias (i.e., *V* = 0).

## 3. Results and Discussion

**Figure 2:**
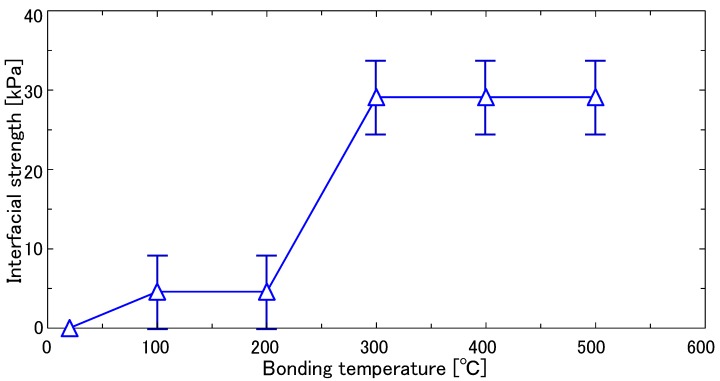
Interfacial bonding strength as a function of temperature for the Si/graphene/Si structure.

**Figure 3:**
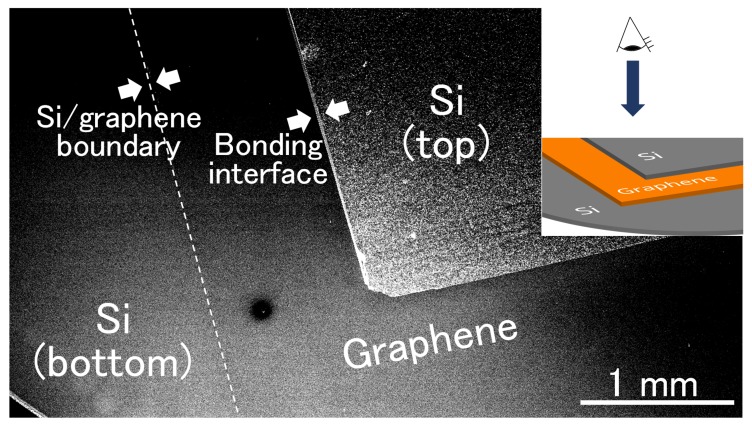
Plane-view scanning electron microscope image of the fabricated Si/graphene/Si double heterostructure.

Interfacial bonding strength as a function of temperature was first analyzed, where the temperature varied from room temperature (20 °C) to 500 °C. Figure 2 shows the dependence of the interfacial bonding strength in the Si/monolayer graphene/Si structure on bonding temperature. For temperatures below 300 °C, an increase in the bonding strength with bonding temperature was observed. This can be attributed to diffusion of residual water on the bonding surfaces into the bulk Si region and/or outgassing from the bonded interface by vaporization. On the contrary, no significant increase in the bonding strength for bonding temperatures above 300 °C was observed, and as a result, the optimum bonding temperature was determined to be 300 °C. Figure 3 shows a plane-view scanning electron microscope image of the fabricated Si/graphene/Si double heterostructure. Here, we have demonstrated success in the preparation of a monolayer-cored double heterostructure composed of Si/graphene/Si.

**Figure 4:**
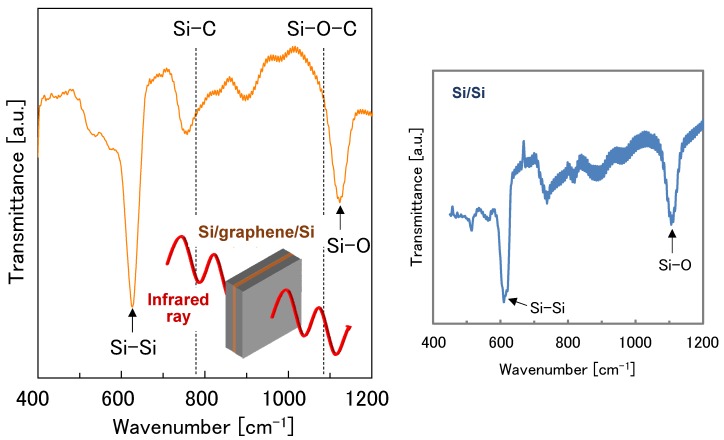
FTIR spectrum of (**left**) the Si/bilayer graphene/Si double heterostructure and (**right**) a Si/Si control structure.

**Figure 5:**
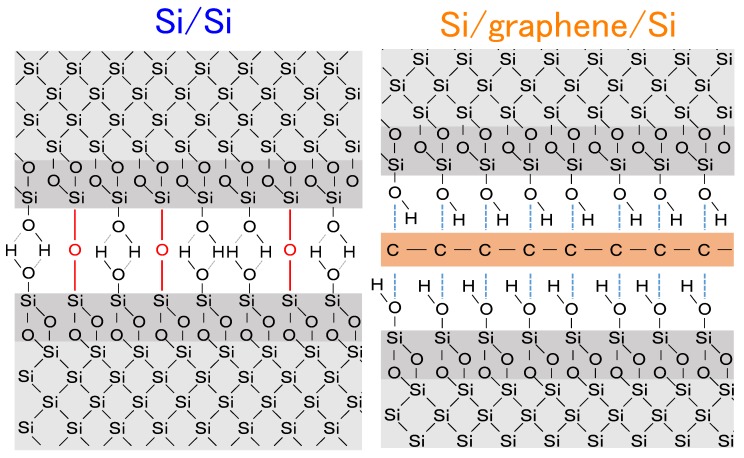
Conceptual schematic of a cross-sectional molecular view of the bonded interfaces: (**left**) Si/Si control structure, (**right**) Si/graphene/Si double heterostructure.

The measured interfacial bonding strength of the Si/graphene/Si structure (~30 kPa) was drastically reduced when compared to the Si/Si control structure without graphene (~650 kPa). To investigate the origin of this interfacial strength difference, an FTIR measurement was conducted on the Si/graphene/Si double heterostructure containing bilayer graphene, as the Si wafer used for monolayer graphene was not transparent. Absorption peaks for covalent Si–C and Si–O–C bonds [31] were not observed in the measurement, as seen in Figure 4. Figure 5 depicts a conceptual schematic of a cross-sectional molecular view of the bonded interfaces. It is known that covalent oxygen bridges are partially formed at the directly bonded Si/Si interface, therefore enhancing the bonding strength [20,21]. Alternatively, when graphene is present, interfacial covalent bonds do not form and the primary bonding forces include van der Waals interactions and hydrogen bonds. These interfacial chemical bonding differences are hypothesized to be the cause for mechanical strength differences in the Si/graphene/Si structure relative to the Si/Si control.

**Figure 6:**
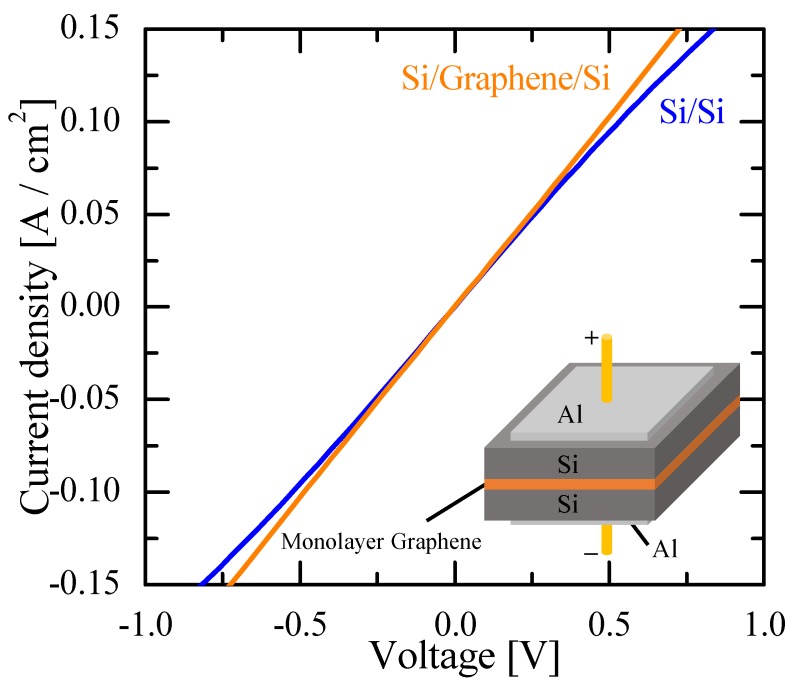
Current-voltage characteristics of the Si/monolayer graphene/Si double heterostructure and corresponding Si/Si control structure with the same doping concentration (~1 × 10^19^ cm^−3^) in the Si wafer.

**Figure 7:**
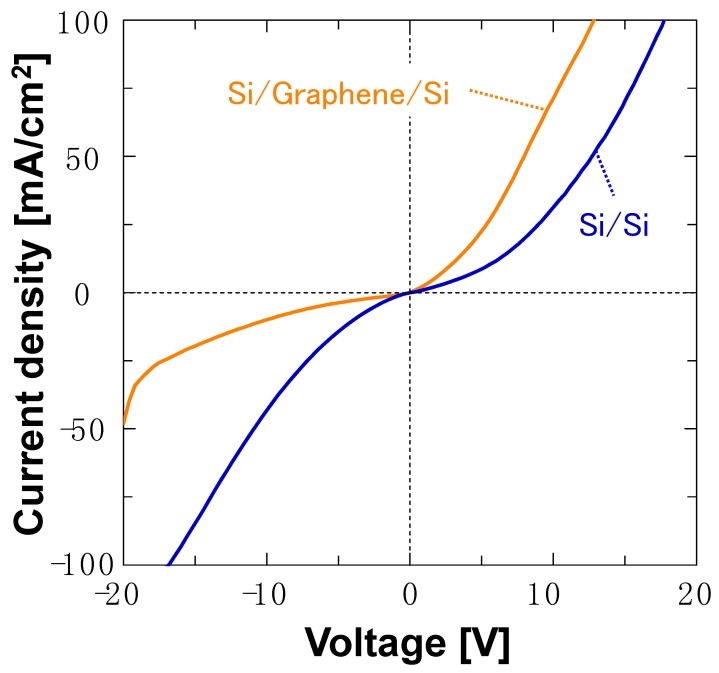
Current-voltage characteristics of the Si/bilayer graphene/Si double heterostructure and corresponding Si/Si control structure with the same doping concentration (~1 × 10^16^ cm^−3^) in the Si wafer.

Figure 6 and Figure 7 show the room-temperature current-voltage characteristics of the Si/monolayer graphene/Si and Si/bilayer graphene/Si double heterostructures, respectively, compared to the Si/Si control sample with the same doping-concentration Si wafer for each. The interfacial electrical resistivity across the Si/monolayer graphene/Si structure was less than 5 Ω cm^2^, and comparable to that for the corresponding Si/Si control, as seen in Figure 6. Thus, a favorable electrical interlayer conductance has been obtained in the bonded Si/graphene/Si double heterostructure. The Si/bilayer graphene/Si double heterostructure exhibited a diode-like, rectified current-voltage curve, similar to that of the corresponding Si/Si control, as shown in Figure 7. This result is consistent with the existing reports that graphene/Si interfaces have Schottky-contact characteristics (for Si with regular doping concentrations) [32,33]. It is hypothesized that the asymmetry in the current-voltage curve, seen in Figure 7, for the Si/bilayer graphene/Si structure may be due to property differences between the two graphene/Si interfaces included in the double heterostructure for our samples. It should be noted that the difference in the electrical characteristics between Figure 6 and Figure 7 may not be due to differences between monolayer and bilayer graphene, but simply due to the difference in doping concentrations in the Si wafers used for each sample. In other words, Si with heavy, degenerating doping concentrations, as seen in the Si wafer used for the Si/bilayer graphene/Si structures here, may form heterointerfaces of the ohmic-contact property with graphene due to its metallic characteristics.

**Figure 8:**
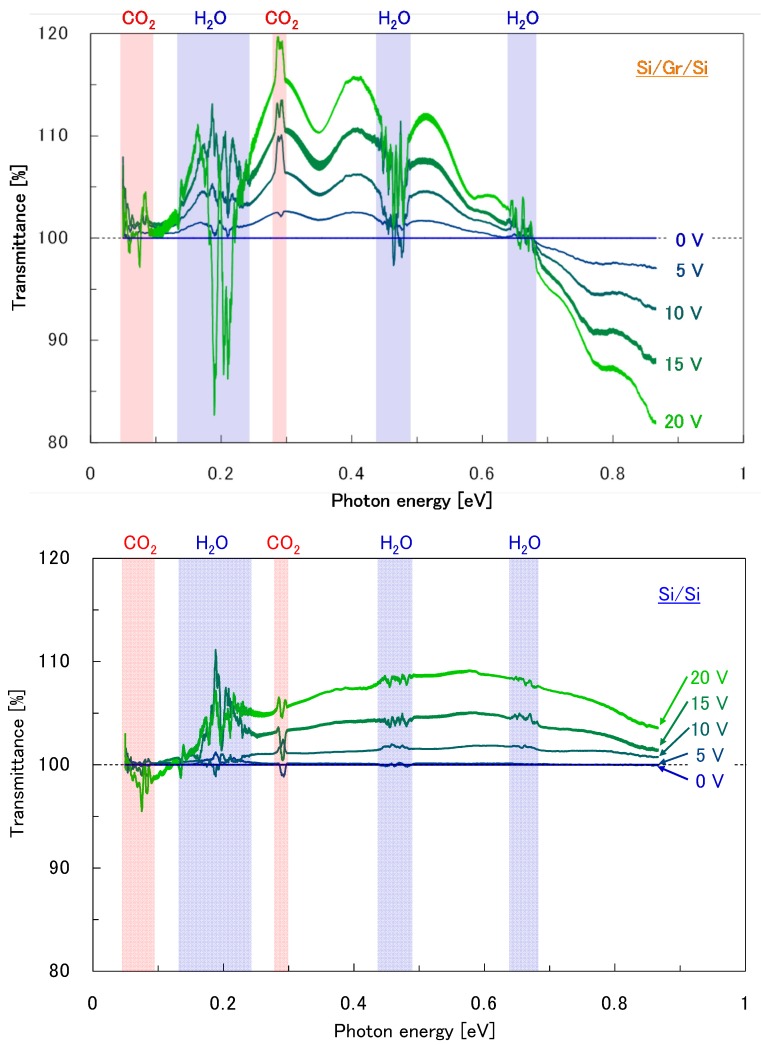
FTIR transmission spectra with applied electrical bias for (**top**) the Si/bilayer graphene/Si double heterostructure and (**bottom**) the Si/Si control sample. The regions highlighted in blue and red contain noise originating from ambient concentration changes in H_2_O and CO_2_, respectively.

Figure 8 shows the FTIR transmission spectra with applied electrical bias for the Si/bilayer graphene/Si double heterostructure as well as the Si/Si control sample. The regions highlighted in blue and red contain noise originating from ambient concentration changes in H_2_O and CO_2_, respectively. As the applied voltage increases, oscillatory changes in the transmittances were observed for both the Si/bilayer graphene/Si and reference Si/Si samples. This spectral oscillation can be attributed to the optical interference at the bonded interfaces, which are magnified due to the existence of interfacial voids. These interfacial voids are known to exist partially at bonded interfaces, originating from airborne particles or partial missing bonds [34,35,36,37], or potentially partial polymer residue used in the graphene transfer process by the vendor, and are presumably expanded by temperature increases induced by the applied electrical bias in the samples.

**Figure 9:**
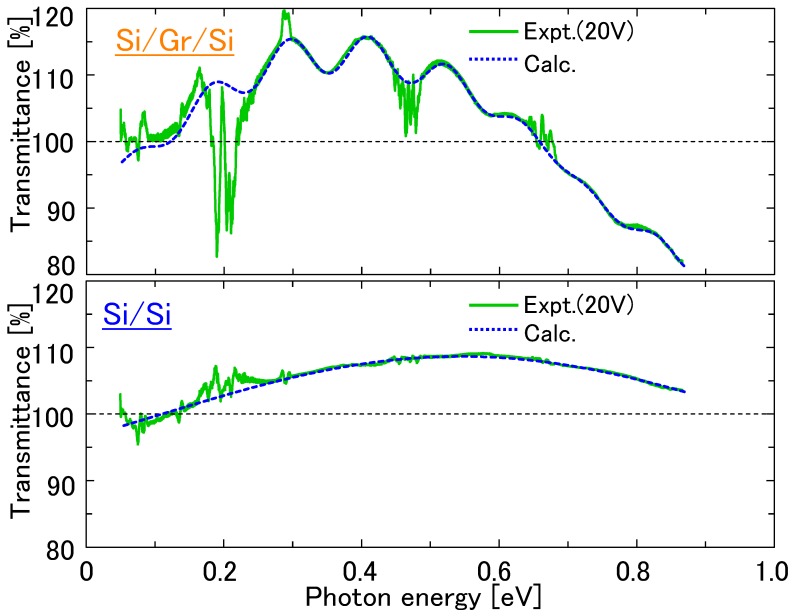
Comparison of the optical interference effect, represented in Equation (1), to the FTIR transmission spectra for (**top**) the Si/bilayer graphene/Si double heterostructure and (**bottom**) the Si/Si control structure at the applied bias voltage of 20 V.

**Figure 10:**
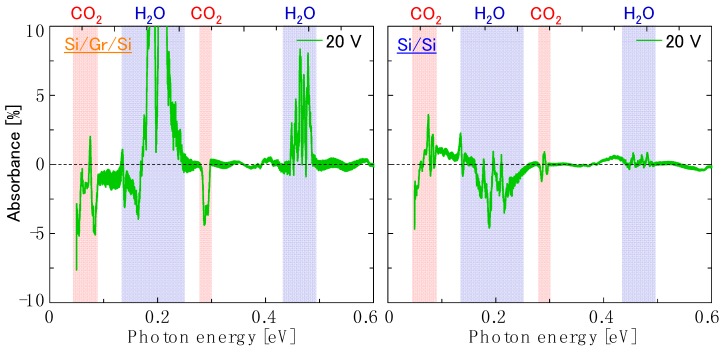
Absorption spectra for (**left**) the Si/bilayer graphene/Si double heterostructure and (**right**) the Si/Si control sample at the applied bias voltage of 20 V, after elimination of the optical interference effect.

For clearer quantitative examination of the transmission spectra, the interference effect was eliminated from the data using a mathematical model for the interference mechanism, as shown below. The change in transmittance is formulated, accounting for the voids of various sizes as:

(1) $$\Delta T=\sum_{k=1}^{m}A_{k}\sin\left\{2\pi \cdot 2nh_{void}\cdot \frac{1}{hc}\left(E_{photon}-\Phi \right)\right\}$$

where *A_k_* is the amplitude, *n* is refractive index, *h_void_* is the void height, *h* is the Planck constant, *c* is the speed of light, *E_photon_* is the photon energy, and Φ is the phase offset. Figure 9 shows the fitting result of Equation (1) to the FTIR transmission spectra for the Si/bilayer graphene/Si and Si/Si structures at the applied bias voltage of 20 V. By subtracting the optical interference effect, obtained as Figure 9, from the original transmission data in Figure 8, attempts were made to clarify the intrinsic absorption by graphene, which is shown in Figure 10. Comparing Figure 10 (left) and (right), there is no significant optical absorption seen in graphene below the level of the atmospheric noise. Thus, we have not been able to observe a signature for graphene bandgap opening, like that reported in Reference [30], for our measurement conditions and sample structure including: Configuration of the metal electrodes, applied bias amplitudes, spectral measurement energy range, and environmental noise level. However, we have presented an experimental scheme for bandgap detection in a planar double heterostructure.

Our fabrication of the Si/graphene/Si structure is the first realization of a monolayer-cored double heterostructure, to the best of our knowledge. Importantly, the choice and use of the chemical elements of carbon (C, graphene) and silicon (Si) presented here have been proven suitable for future device use due to their low cost, earth abundance, and environmental friendliness. The Si/graphene/Si double heterostructures presented here can form a new basis for the next-generation nanophotonic devices with superb optical and electrical properties provided by the graphene [3,7,9], and high photon, carrier confinements, and excellent optical and electrical controllability provided by silicon clads. We would like to stress that a great advantage of the wafer bonding technique is its capability to fabricate double heterostructures that are not formable by conventional growth methods. We hereby realized a monocrystalline Si/graphene/monocrystalline Si double heterostructure, unable to be constructed by epitaxy, by utilizing wafer bonding. Our fabrication scheme and structure provide a new device platform for high-efficiency nano-optoelectronics. The results presented here are the first realization/demonstration of semiconductor wafer bonding mediated by an atomic monolayer, to the best of our knowledge. There exists potential for alternate monolayer materials, including graphene, to also be suitable as mediating agents for semiconductor bonding in optoelectronic applications, such as multijunction solar cells, due to their high adhesion strength [38], thermal and electrical conductivity [3,7,9], optical transparency [39], and environmental friendliness. Incorporation of a Si etch-back [40,41] or ion-cutting [42,43,44] technique, or the use of commercial silicon-on-insulator wafers [19,45,46] with HF separation, would enable the production of thin-film devices comprising Si/graphene/Si double heterostructures for lightweight, flexible, and ambient optoelectronics [47,48,49,50,51] composed of environmentally and human-body friendly chemical elements (C, Si).

## 4. Conclusions

In this work, we have fabricated, for the first time, a monolayer-cored double heterostructure, towards the realization of high-efficiency nano-optoelectronic devices. We prepared a Si/graphene/Si stack by means of graphene-mediated wafer bonding and verified the interfacial mechanical stability and interlayer electrical connection, demonstrating a new application of wafer bonding. The fabrication scheme and structure presented here provides a new device platform for functional monolayer materials.
